# Fatty Liver Disease, Metabolism and Alcohol Interplay: A Comprehensive Review

**DOI:** 10.3390/ijms24097791

**Published:** 2023-04-24

**Authors:** Aitor Odriozola, Alvaro Santos-Laso, María del Barrio, Joaquín Cabezas, Paula Iruzubieta, María Teresa Arias-Loste, Coral Rivas, Juan Carlos Rodríguez Duque, Ángela Antón, Emilio Fábrega, Javier Crespo

**Affiliations:** Gastroenterology and Hepatology Department, Clinical and Translational Research in Digestive Diseases, Valdecilla Research Institute (IDIVAL), Marqués de Valdecilla Universitary Hospital, Av. Valdecilla 25, 39008 Santander, Cantabria, Spain

**Keywords:** non-alcoholic fatty liver disease, alcohol-related liver disease, cirrhosis, hepatocellular carcinoma

## Abstract

Non-alcoholic fatty liver disease (NAFLD) is the most common cause of chronic liver disease worldwide, and its incidence has been increasing in recent years because of the high prevalence of obesity and metabolic syndrome in the Western population. Alcohol-related liver disease (ArLD) is the most common cause of cirrhosis and constitutes the leading cause of cirrhosis-related deaths worldwide. Both NAFLD and ArLD constitute well-known causes of liver damage, with some similarities in their pathophysiology. For this reason, they can lead to the progression of liver disease, being responsible for a high proportion of liver-related events and liver-related deaths. Whether ArLD impacts the prognosis and progression of liver damage in patients with NAFLD is still a matter of debate. Nowadays, the synergistic deleterious effect of obesity and diabetes is clearly established in patients with ArLD and heavy alcohol consumption. However, it is still unknown whether low to moderate amounts of alcohol are good or bad for liver health. The measurement and identification of the possible synergistic deleterious effect of alcohol consumption in the assessment of patients with NAFLD is crucial for clinicians, since early intervention, advising abstinence and controlling cardiovascular risk factors would improve the prognosis of patients with both comorbidities. This article seeks to perform a comprehensive review of the pathophysiology of both disorders and measure the impact of alcohol consumption in patients with NAFLD.

## 1. Introduction

Liver diseases can occur as a consequence of multiple etiological factors. End-stage liver disease, mainly cirrhosis and/or hepatocellular carcinoma (HCC), is responsible for the vast majority of liver-related deaths. In this context, non-alcoholic fatty liver disease (NAFLD) and alcohol-related liver disease (ArLD) are the most important causes of liver damage [1,2]. The former, is the most common liver disease worldwide, with an estimated prevalence between 25% and 40% in adults, and incidence is increasing because of the great prevalence of obesity and metabolic disorders among the general population [3]. The latter was found to be the main cause of all liver cirrhosis-related deaths worldwide [4] by the Global Burden of Disease (GBD) project, responsible for almost 50% [5].

The NAFLD spectrum ranges from simple steatosis, to being determinant in liver disease progression, to non-alcoholic steatohepatitis (NASH), which is implicated in the development of fibrosis, cirrhosis and HCC [6]. The ArLD spectrum ranges from alcohol-related fatty liver (ArFL) to alcohol-related steatohepatitis (ArSH) in 10–35% of individuals who continue with chronic heavy alcohol consumption for years. Moreover, between 8% and 20% of patients with alcohol-related steatohepatitis (ArSH) and chronic heavy alcohol consumption will develop alcohol-related liver cirrhosis and HCC [7]. Acute ArLD, called alcohol-related hepatitis (AH), is present in patients with severe acute ArSH, jaundice and liver failure [7,8,9].

As previously described, fatty liver hepatic morphology constitutes a prerequisite for both pathologies. Furthermore, histological findings for ArSH and NASH look similar, suggesting common pathogenic mechanisms are implicated in the disease progression.

This article aims to perform a comprehensive review of the pathophysiology, molecular features, and medical challenges of the effect of alcohol intake on patients with non-alcoholic fatty liver disease. It constitutes a relevant co-factor for liver damage in NAFLD, increasing the risk of disease progression with the development of more severe inflammation, fibrosis, cirrhosis, and HCC.

## 2. The Key Aspects in NAFLD

### 2.1. From NAFLD to Metabolic-Associated Fatty Liver Disease (MAFLD)

NAFLD is characterized by excessive hepatic fat accumulation and insulin resistance (IR) in the absence of both secondary causes of liver damage and of a daily consumption >30 g for men and >20 g for women [10]. The presence of inflammation due to fat accumulation (NASH) constitutes a clinically relevant feature because of its implication in the development of fibrosis and cirrhosis. Prevalence of NAFLD is estimated to be between 25% and 40% worldwide [3]. Nearly 25% of these NAFLD patients will progress to NASH [11,12]; the reasons why only some of these patients progress to NASH is not well understood, but obesity and IR seem to be involved [13].

Increased incidence of obesity and metabolic syndrome in the Western population has become a relevant public health issue as a consequence of increasingly unhealthy diets with excess calorie intake as well as sedentary behaviors. Moreover, the lack of “positive” criteria for diagnosis of this disease when not due to alcohol has led to newly proposed nomenclature, namely metabolic dysfunction-associated fatty liver disease (MAFLD), which appears to be a more appropriate term [14]. In line with this change, exclusion of concomitant liver diseases in patients who meet the criteria for MAFLD is no longer a prerequisite, and dual etiology is widely accepted.

### 2.2. Histological Features of NASH

Diagnosis of NASH requires a liver biopsy to establish the difference between non-alcoholic fatty liver (NAFL) and other causes of liver damage. Sampling variability must be taken into account to avoid misdiagnosis and staging inaccuracies [15].

Histological features of NASH comprise the simultaneous presence of steatosis (>5% hepatocytes), ballooning (as a result of hepatocyte damage) and lobular inflammation. When one of the three components defining NASH is not present, NAFLD diagnosis must be done [16,17,18]. Other findings are present in NASH but are not considered for diagnostic criteria: Mallory–Denk bodies, portal inflammation, megamitochondria, polymorphonuclear infiltrates, apoptotic bodies, clear vacuolated nuclei and microvacuolar steatosis [10].

The term steatohepatitis was first proposed in a Mayo Clinic study aiming to characterize the relationship between severity and zonal location of steatosis in NASH patients [19]. Earlier, alcoholic-like findings in liver biopsies of overweight and/or diabetic patients were described in two studies by Zimmerman [20,21].

Until recently, only one pattern was recognized to be associated with the development of advanced fibrosis, defined as a zone 3 centered injury pattern that includes steatosis, inflammation, and ballooning in variable degrees with or without fibrosis [18].

However, a previously unrecognized pattern of fibrotic fatty liver disease in children in which the injury involves acinar zone 1 rather than zone 3 has demonstrated a potentially fibrotic progressive form of NAFLD without the full spectrum of features of steatohepatitis. It is denominated as “borderline, zone 1, steatohepatitis” [22].

Finally, “burned-out NASH” refers to the regression of typical findings in NASH diagnosis in the more advanced grades of the disease [10].

### 2.3. Multiple Parallel Hits in NAFLD

The pathophysiology of NAFLD is complex and incompletely understood. A recent theory has been proposed to explain the pathophysiology of liver damage in NAFLD in which “multiple parallel hits” derived from the gut and/or the adipose tissue (AT) may promote liver inflammation [23]. In this hypothesis, several molecular mediators derived from various organs may be implicated in triggering inflammation, which may later progress to fibrosis and carcinogenesis.

### 2.4. Adipose Tissue Inflammation

Nowadays, AT is recognized as key in fatty acid metabolism and homeostasis due to its role in mediating immune response through the excretion of different chemokines and cytokines. In healthy individuals, AT interacts with the liver to control energy homeostasis [23]. Other situations, such as obesity or metabolic syndrome, promote local inflammation through the secretion of several mediators from AT, which increase and perpetuate systemic inflammation and deteriorate liver function. This interaction between AT and the liver is known as the AT–liver axis interaction [23,24].

Primary features in AT inflammation are still a matter of debate, but diet-induced stress due to high-fat diets (HFDs), sugars and excessive calorie intake may induce a chemokine and cytokine response with subsequent immune cell infiltration of AT. This cell infiltration is the consequence of interaction between innate immune cells (e.g., macrophages) and adaptative immune response cells (e.g., T-cells). The former is dependent on chemokines such as C-C motif chemokine ligand 2 (CCL2) excreted from T-cells [25]. The latter are recruited to AT by antigen presentation and increase the expression of various chemokines such as CCL13 and CCL5, which are increased in obese patients [26].

Once the local inflammation is established through the infiltrate of immune cells, this response is regulated and perpetuated by the expression of the key adipocytokines in AT: tumor necrosis factor alpha (TNFα), interleukin-1-beta (IL-1β) and interleukin-6 (IL-6) [27]; and the misexpression of anti-inflammatory adipocytokines: adiponectin and leptin [28,29,30].

### 2.5. TNFα, IL-1β and IL-6

These cytokines are thought to play a key role in the pathophysiology of NAFLD and NASH through the amplification of the immune response, promoting the interaction between AT and the liver and increasing the systemic inflammation state, which will exert its deleterious effect on liver function.

According to this hypothesis, both TNFα and IL-6 have an increased expression in human fat cells of obese patients [31,32]. In addition, weight loss in this population results in decreased IL-6 [33] and TNFα [34], reflecting its involvement in NAFLD development.

Furthermore, other chemokines, whose expression is mediated by the secretion of IL-6 from AT, such as JNK1, are involved in the development of hyperinsulinemia, hepatic steatosis and hepatic IR, demonstrating AT-derived IL-6 regulated distal metabolic effects on the liver [35]. In fact, IL-6 has been demonstrated to be expressed up to 100-fold higher in AT than in the liver in obese patients, suggesting that AT is the main source of this cytokine [36]. SOC3, a hepatic chemokine whose expression is mediated by IL-6 and TNFα, is responsible for the regulation of hepatic insulin resistance. Decreased levels of IL-6 and TNFα in the context of weight loss have shown a downregulation of this chemokine, with the consequent improvement in hepatic insulin sensitivity [37].

### 2.6. Adiponectin and Leptin

Both adiponectin and leptin are anti-inflammatory adipocytokines involved in fatty acid oxidation and regulation of fat content in several organs [28,29,30,38].

Adiponectin levels increase after weight loss and are decreased in obesity [29]. This cytokine exerts its effect by activating adenosine monophosphate-activated protein kinase (AMPK) and Sirtuin 1 (Sirt1) [39]. Downregulation or deletion of Sirt1 has been related to hepatic steatosis, endoplasmic reticulum stress and liver inflammation [40].

Leptin secretion increases proportionally to triglycerides in order to reduce the fat content in peripheral organs through fatty acid oxidation [41,42]. This oxidative capacity is fully operative in the liver, minimizing ectopic lipid accumulation in this organ. However, its implication in NAFLD development is not known.

### 2.7. Gut Microbiome (GM)

GM interacts with the liver via the so-called “liver–gut axis” [43,44,45]. Increased permeability of the mucosa and gut microbial imbalance leads the bacterial metabolism products and the pathogen-associated molecular patterns (PAMPs) to reach the liver via portal circulation, activating several inflammation pathways and leading to liver injury and fibrosis [46,47,48].

GM can also lead to liver injury through the interaction between bacterial byproducts and several glucose and lipid metabolism pathways, which will increase insulin resistance and hepatic steatosis [49,50]. Modulation of bile acids (BAs) by GM [51] has been proposed to play a key role in triggering metabolic diseases such NAFLD [52].

As previously mentioned, those bacterial metabolites that cross the intestinal barrier may provide a benefit for health (e.g., regulating immunity) [53,54], while others may deregulate intestinal permeability and BA metabolism, causing liver damage [55]. These metabolites are the byproducts of numerous pathways.

### 2.8. Microbial Fermentative Pathways

Metabolic function of the microbiota may provide increased levels of some metabolites, which can play a key role in the development of NAFLD. High alcohol production by the microbiota has been reported in several studies in patients with NAFLD [56,57,58].

When ethanol and its metabolites reach the liver, many metabolic pathways are upregulated. For example, alcohol dehydrogenase concentrations increase in order to convert ethanol into acetaldehyde and acetate. These metabolites have been implicated in weakening intestinal tight junctions, increasing intestinal permeability and enabling translocation of microbial byproducts [59,60,61]. Metabolization of ethanol in the liver has been implicated in the formation of free fatty acids and oxidative stress.

Patients with NAFLD show increased levels of alcohol dehydrogenase, aldehyde dehydrogenase and catalase, which are upregulated in the NASH liver, suggesting alcohol is a key factor in triggering NAFLD pathogenesis. In fact, there is evidence of some strains of bacteria with high alcohol production inducing NAFLD [62,63].

It is not only ethanol that derives from the metabolism of GM. Short-chain fatty acids (SCFAs) such as acetate, propionate and butyrate are produced as a result of the fermentation of complex carbohydrates (from dietary fiber) by the gut microbiome. Most are consumed in the gut, but some reach the liver, taking part in metabolic pathways such as gluconeogenesis and lipogenesis, and increasing insulin resistance and AT inflammation [64,65].

Recently, Crespo et al., proposed a theory in which endogenous ethanol produced by the gut microbiome may be implicated in the development and progression of NASH, showing its similarities with alcoholic fatty liver disease and “auto-brewery” syndrome. The latter constitutes an example of how alterations in microbial composition may cause a clinical syndrome of alcohol intoxication after ingesting carbohydrate-rich meals. A less severe situation, where enough ethanol may be produced to cause chronic damage to the liver, has been proposed to occur in NASH [66]. Furthermore, it has recently been demonstrated that the first-pass effect of the liver obscures the levels of endogenous ethanol production, suggesting that microbial ethanol could be considered in the pathogenesis of this highly prevalent liver disease [67].

### 2.9. Other Pathways

Choline is a quaternary ammonium alcohol that plays a key role in liver fat metabolism. In the liver, choline is converted into phosphatidylcholine (lecithin), an essential component of cell membranes, which prevents hepatic accumulation of triglycerides through the excretion of VLDL particles. In the gut, choline can be converted into trimethylamine (TMA) by intestinal bacteria. In the liver, TMA is oxidized by the enzyme flavin mono-oxygenase 3 (FMO3) to generate trimethylamine-N-oxide (TMAO), which has been implicated in the development of atherosclerosis and obesity. The altered microbiome produces increased activation of the TMAO pathway and decreased production of lecithin, favoring a pro-inflammatory environment in the liver, atherosclerosis, AT inflammation and insulin resistance [68,69].

### 2.10. Genetics

The spectrum of fatty liver is wide, and the fact that not all patients with fatty liver develop inflammation suggests the possibility of additional factors contributing to this evolution.

Although unlikely, NAFLD has been proposed to be a heritable disorder. In this way, polymorphisms in patatin-like phospholipase 3 (PNPLA3), encoding a protein with homology to lipid acyl hydrolases (adiponutrin), have been associated with higher levels of hepatic steatosis in NAFLD [70,71,72,73]. A loss of function in the enzyme due to I148M substitution results in increased lipid droplets of triglycerides and retinyl esters in hepatocytes and hepatic stellate cells, inducing liver damage and fibrosis [73,74].

Homozygosity for PNPLA3 I148M polymorphism is associated with the degree of inflammation and liver fibrosis. In contrast, in vivo studies showed the absence of fatty liver, normal values of aminotransferases and no evidence of insulin resistance in PNPLA3-deficient mice.

Transmembrane 6 superfamily member 2 (TM6SF2) transfers triglycerides to apolipoprotein B100 from the hepatocyte. The rs58542926 C > T polymorphism results in a loss of function with hepatic fat accumulation and lower circulating lipoproteins [75,76,77].

Membrane-bound O-acyltransferase domain-containing 7 (MBOAT7) is a protein that remodels phosphatidylinositol with arachidonic acid. Downregulation of MBOAT7 at the mRNA and protein level reduces this metabolite in hepatocytes and circulation. This fact has been linked with the risk of NAFLD, inflammation, fibrosis, and progression of NAFLD to HCC [78,79,80].

The glucokinase regulator (GCKR) regulates de novo lipogenesis through the influx of glucose in hepatocytes. A loss of function of this receptor results in hepatic fat accumulation secondary to increased lipogenesis, stimulated by high levels of glucose in the hepatocyte [81,82,83].

### 2.11. Endoplasmic Reticulum (ER) Stress

Misfolded proteins in the ER are the consequence of an imbalance between energy supply and demand, frequently observed in obesity and metabolic syndrome. They originate cellular stress and activate the unfolded protein response (UPR). The UPR is mediated by at least three stress-sensing pathways: pancreatic ER kinase (PERK), inositol-requiring enzyme 1 (IRE1) and activating transcription factor 6 (ATF6). When this occurs, transcription factor XBP1 is activated, and it regulates lipid synthesis and inflammatory cascades at various stages: (1) IRE1-mediated activation of JNK; (2) activation of IKK-NF-kβ signaling pathways; (3) production of reactive oxygen specimens (ROS) [84,85,86]. These pathways are involved in lipid synthesis/accumulation, leptin resistance, adipogenesis, inflammation, and insulin signaling/resistance [87,88].

### 2.12. Epigenetics

Epigenetic factors have been postulated to play a key role in modulating the individual susceptibility to NAFLD. In fact, intrauterine exposure to high-fat diet (HFD) in mice worsened visceral fat accumulation and insulin resistance when mice were re-fed with an HFD after birth, and also resulted in increased steatosis and higher risk of NASH [89]. These changes are the result of the reduction of hepatic mitochondrial electron transport chain (ETC) enzyme complex activity and the upregulation of oxidative stress genes (Nos3, Nos2, Gstm6, and Lcn2), inflammation genes (Crp, Mmd2, Tnfsf1, and Il-12β) and genes involved in cardiolipin (Pgp), fatty acid (Acl, Acacb, Fas, Srebp1c), and triacylglycerol (TAG) synthesis (Gpam, Agpat, Lpp2, Dgat1).

For these reasons, epigenetic changes modulate and interact with inherited risk factors to determine the individual susceptibility to NAFLD and NASH development, promoting new targets for its management and treatment.

**Figure 1:**
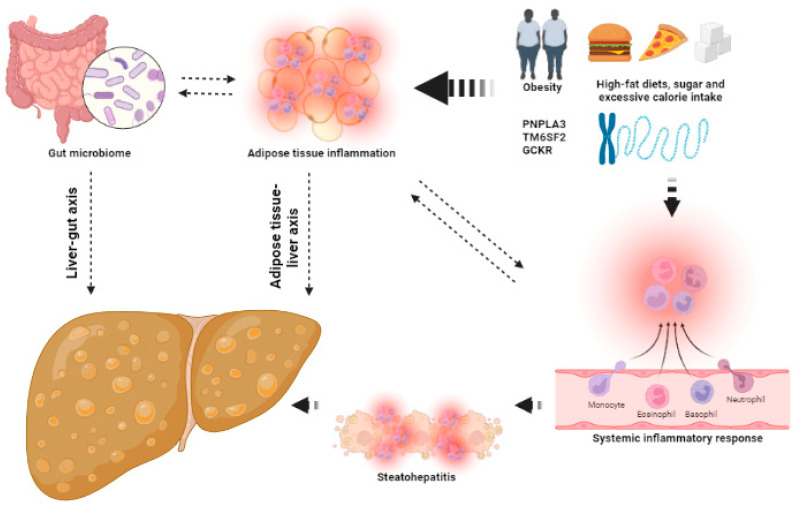
The keys aspects of the pathophysiology of NAFLD. Several factors, such as obesity, high-fat diets and (epi)genetics, lead to adipose tissue inflammation and dysbiosis, increasing systemic inflammatory response and leading to the development of hepatic steatosis and steatohepatitis. This image has been created using BioRender.

The mechanisms that contribute to the development of NAFLD are shown in Figure 1.

## 3. The Key Aspects in Alcohol-Related Liver Disease (ArLD)

### 3.1. Proposed Threshold of Alcohol Consumption for Increasing the Risk of ArLD

The definition of chronic heavy alcohol consumption involves the intake of >40 g of pure alcohol per day (equating to 375 mL of 13 vol% wine or >1 L of 5 vol% beer) over a sustained period of time (frequently, years) which leads to the highest risk of ArLD [90].

However, it has been demonstrated that chronic consumption of 12–24 g of alcohol per day increases the risk of cirrhosis compared with non-drinking, so lower levels of alcohol intake may lead to increased risk of ArLD and can be more difficult to detect [91].

A major clinical problem appears given that the typical serving size of drinks varies among countries. These variants establish a standard drink equaling 8 g of pure alcohol in the UK, 10 g in the USA, 19.75 g in Japan and 10 g in Europe. Moreover, recommendations of what constitutes “heavy” drinking also vary from country to country. In the UK, alcohol consumption beyond 112 g per week in both men and women is not recommended [92]. The USA recommends no more than 42 g and 56 g per day for women and men (<66 years) with weekly limits of 98 g and 196 g, respectively. Finally, in Japan amounts beyond 20 g per day in women or 40 g per day in men are not recommended.

Due to these differences in what constitutes “too much” alcohol among countries, it is assumed that a standard drink contains 10 g of alcohol, with this quantity being approximately the mean average amount that is recognized to lead to health issues in countries worldwide. For a more precise study of the impact of alcohol consumption, we should start using alcohol biomarkers [93], which are useful tools to monitor abstinence.

### 3.2. Histological Features of ArLD

The histological spectrum of ArLD includes ArFL, ArSH, alcoholic fibrosis and/or cirrhosis and HCC.

In the case of ArFL, large lipid droplets in the hepatocytes constitute the main finding. This macrovesicular steatosis displaces the nucleus towards the plasma membrane and does not show any sign of inflammation.

ArSH is defined by the appearance of hepatocellular injury, including ballooning and Mallory–Denk bodies, necrosis, lobular inflammation with mononuclear and neutrophilic granulocytes, and variable macrovesicular (with microvesicular) steatosis within hepatocytes. These findings are comparable to those reported in NASH. When fibrosis appears, perivenular fibrosis and fibro-obliterative changes of venous vessels are typical, linking central veins and portal tracts in septal configuration [94,95].

In severe cases of ArSH, cholestasis may be present in hepatocytes, canaliculi, and bile ducts. Apparition of extensive microvesicular steatosis, cholestasis and fibro-obliterative damage of venous vessels of the liver has not been demonstrated in NAFLD.

Furthermore, liver histology across ArLD is not only useful for diagnosis but also has prognostic value. In fact, few scoring systems have been developed in order to stratify and assess the severity of ArLD in a reproducible and prognostically relevant manner [96,97].

### 3.3. Pathophysiology of ArLD

ArLD can be caused by chronic consumption of alcohol exceeding daily amounts, which vary among different individuals. For this reason, it is supposed that several factors may modulate the individual susceptibility to ArLD, independently of the daily amount of alcohol consumption. Epigenetics, metabolic alterations, oxidative stress, and inflammation contribute to ArLD, affecting hepatocytes and hepatic stellate cells.

### 3.4. Genetics

Genetic factors have been demonstrated to predispose to both alcohol use disorder (AUD) and the development of ArLD [98,99].

Genome-wide association studies revealed PNPLA3, TM6SF2 and MBOAT7 as the main genetic determinants of ArLD. As previously described, PNPLA3 is involved in lipid metabolism (also the main known genetic risk factor for NAFLD), TM6SF2 results in hepatic fat accumulation and MBOAT7 causes a disturbance in the acetylation of phosphatidylinositol. These changes have been related to hepatic steatosis, inflammation and risk of fibrosis, which will determine the degree and risk of ArLD. Moreover, a synergistic effect of alcohol consumption in patients with NAFLD with these genetic variants has been demonstrated. Patients with NAFLD and these genetic variants show enhanced inflammation processes with the addition of a second factor such as alcohol, via dysfunctional lipid turnover between phospholipids and lysophospholipids [100,101,102].

Other minority gene alterations have been proposed to predispose to ArLD. Genes encoding inflammatory mediators (TNFα and IL-1), genes involved in the endotoxin response (CD 14 endotoxin receptor) and genes involved in oxidative stress (glutathione-S-transferase and manganese superoxide dismutase) have been related to an increased individual susceptibility to ArLD [103].

### 3.5. Oxidative Stress

Liver metabolism is characterized by two main pathways: oxidative and non-oxidative metabolism. Oxidative metabolism is exerted by alcohol-dehydrogenase and the CYP2E1 enzyme. The former metabolizes ethanol through alcohol dehydrogenase into acetaldehyde in hepatocytes. The latter consumes oxygen (increasing levels of reactive-oxygen species, ROS) and NADPH in order to also produce acetaldehyde. CYP2E1 is induced and upregulated in chronic alcohol consumption [104,105].

Acetaldehyde, the product of both metabolic pathways, is toxic and leads to mitochondrial alterations, including decreased ATP generation via the respiratory chain and the production of ROS in the hepatocytes. In addition, alcohol consumption has toxic effects by itself and causes oxidative stress, which is mediated through the generation of ROS [106].

ROS alters the functional properties of many proteins, generates neoantigens and binds directly to DNA, leading to liver damage, inflammation, and fibrosis. Lipid peroxidation, mediated by ROS, produces 4-hydroxynonenal (4-HNE) and malondialdehyde (MDA), which bind to DNA bases and exert a carcinogenic effect in many organs, including the liver [107,108,109,110].

### 3.6. Epigenetics

Epigenetic changes can be induced in the liver by alcohol [111], leading to dysregulated hepatocytes and immune cell functions [112]. One of the key findings in this setting is the downregulation, induced by alcohol, of SIRT1, which results in the upregulation of sterol regulatory element-binding protein 1 (SREBP1) and a subsequent decrease in hepatic lipid metabolism, leading to fatty liver [113,114]. An hypomethylation of DNA (up to 40% in rats after intragastric alcohol feeding) has been demonstrated in ArLD, leading to transcriptional activation and alteration of cellular function [115]. Immune cell functions are also altered in this context through the increased activity of HDAC11, which decreases the production of anti-inflammatory IL-10 [116].

### 3.7. Steatosis in the Setting of ArFL

Alcohol can induce hepatic steatosis through several mechanisms. The main process for the development of hepatic steatosis and AFL comprises alterations in fat metabolism [117,118]. In this way, alcohol increases the proportion of reduced NAD (NADH) in hepatocytes, which inhibits mitochondrial β -oxidation of fatty acids and leads to steatosis. Moreover, upregulation of SREBP1 and inhibition of peroxisome proliferator-activated receptor-α (PPARα, which upregulates many genes involved in free fatty acid transport and oxidation) contribute to the development of hepatic fat accumulation [119,120].

Finally, some mechanisms implicated are independent of alterations in fat metabolism. Acetate, derived from acetaldehyde, can be converted to acetyl-CoA, which contributes to fatty acid synthesis. Alcohol consumption induces lipolysis and adipocyte death, increasing circulating fatty acids with its subsequent hepatic accumulation. Increased supply of lipids from the small intestine to the liver has also been demonstrated to be a co-factor of steatosis development [121].

### 3.8. Inflammation: From ArFL to ArSH

The transition from fatty liver to the appearance of inflammation is the key point that most impacts prognosis due to its implications in the development of fibrosis, cirrhosis and HCC. This progression is driven through the alterations derived from chronic alcohol consumption, which consist of gut-derived PAMPs that release cytokines and chemokines from Kupffer cells, and damage-associated molecular patterns (DAMPs) released by dying hepatocytes. Furthermore, the adaptative immune response activated by neoantigens (as a result of protein adducts with acetaldehyde and ROS) may contribute to inflammation [122].

The inflammation triggered by PAMPs and DAMPs is mediated by pro-inflammatory cytokines such as TNFα, IL-6 and IL-1 β. These molecules perpetuate and amplify the inflammatory response, which leads to liver damage and, subsequently, hepatic fibrosis [123,124,125].

Other molecules could play a role in the development of inflammation in ArLD. In fact, chronic alcohol consumption increases the expression of miR-155 in Kupffer cells. This miRNA has been related with increased concentrations of TNFα, contributing to trigger the inflammatory cascade in the liver [126,127,128,129].

Inhibition of the ubiquitin-proteasome pathway has been proposed to contribute to ArSH through the alteration of cell cycle checkpoints and the activation of transcription factors (NF-kB and hypoxia-inducible factor 1α (HIF1α)) [130] that lead to cellular injury, proliferation and apoptosis. This effect is mediated by the stabilization of abnormal proteins normally degraded by proteasomes, which constitute a pro-inflammatory stimulus that increases the transcription of inflammation mediators, inducing ER stress [131].

An acute phenotype of liver inflammation mediated by alcohol consumption, known as alcohol-related hepatitis (AH) [132], leads to decreased liver function, with increased bilirubinostasis, severe fibrosis and ductular reaction, compared to the case of non-decompensated ArLD [133] Several mediators have been related to the increased inflammatory response that leads to alcohol-related hepatitis. For example, chemokine CCL20 upregulation is closely related to LPS and may not only be a novel potential biomarker to predict disease progression in patients with AH but also an important mediator linking liver inflammation, injury and fibrosis [134]. Toll-like receptor 7 (TLR7), an endosomal TLR that is activated by single-stranded RNA, including endogenous microRNAs linked to ethanol consumption, may contribute to the increased inflammatory response associated with AH through its endogenous ligand let-7b [135].

It is important to note that in AH patients, ductular cell expansion correlates with portal hypertension and collagen expression. This reaction is mediated by LPS-TLR4. For this reason, interventions aimed at lowering serum LPS levels in AH patients might have beneficial effects by preventing the development of a ductal reaction [136]. Furthermore, HNF4α P2 upregulation mediated by TGFβ1 in the context of AH has been identified as a key mediator that results in defective metabolic and synthetic function in hepatocytes, leading to severe forms of AH and emerging as a possible target to improve hepatocellular function in patients with AH [137]. As mentioned before, the degree of cholestasis is an important disease driver in AH. Serum levels of conjugated bile acids are significantly increased in patients with AH, followed by an increase in FGF19 (a major regulator of bile acid synthesis) and a decrease in de novo bile acid synthesis [138].

Excessive alcohol consumption is associated with dysregulation of the microbiome in patients with alcohol use disorder (AUD). However, how the microbiome responds when patients stop drinking has not been well characterized. Few studies have provided insights into the link between functional alterations of the gut microbiota [139], intestinal virome [140] and intestinal candidas [141] with steatosis and inflammation associated with alcohol consumption, emerging as therapeutic targets in ArLD [142].

**Figure 2:**
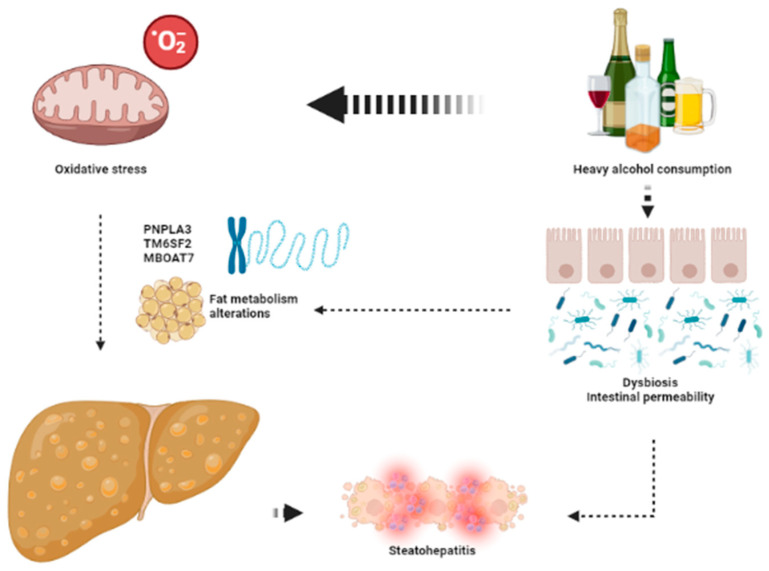
The key aspects in the pathophysiology of ArLD. Heavy alcohol consumption exerts its deleterious effects through direct mitochondrial toxicity leading to oxidative stress and dysbiosis that favors development of liver steatosis and steatohepatitis. This image has been created using BioRender.

The keys aspects leading to the development of ArLD are shown in Figure 2.

## 4. Influence of Alcohol in NAFLD

### 4.1. Overlap in Pathogenesis of ArLD and NAFLD

**Figure 3:**
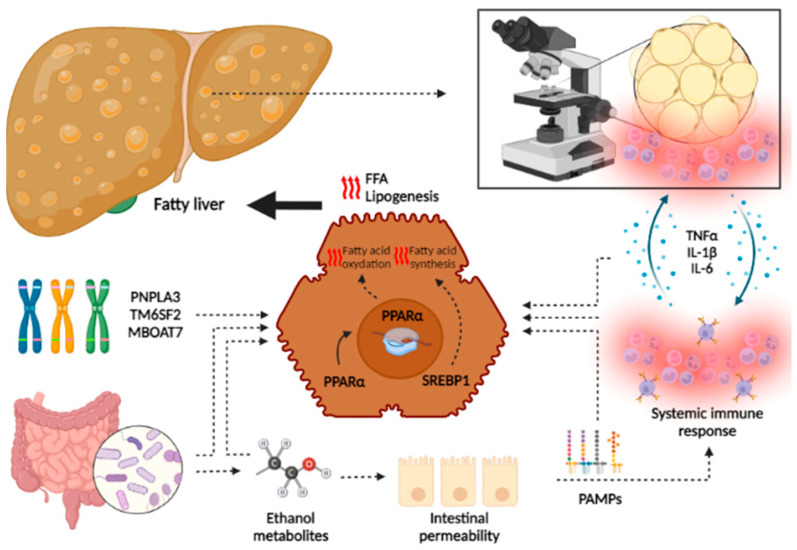
Overlapping in pathophysiology of liver damage in NAFLD and ArLD. Although specific pathways are involved in the development of both NAFLD and ArLD, this figure shows the common processes that lead to hepatic fat accumulation and inflammation. In both pathologies, genetic predisposition leads to the upregulation of several pathways that increase lipogenesis and oxidative stress. Moreover, gut synthesis of ethanol metabolites (by gut microbiome in the case of NAFLD or by alcohol intake in ArLD) leads to an increased intestinal permeability and translocation of PAMPs that activate systemic immune response, increasing the synthesis of several inflammation mediators. These molecules lead to hepatic fat accumulation and the apparition of an immune infiltrate that leads to progressive liver damage. Steatohepatitis also exerts positive feedback, stimulating immune response and increased systemic inflammation. The following abbreviations should be noted: FFA free fatty acid; IL: interleukin, PAMPs: pathogen-associated molecular patterns; PPAR: peroxisome proliferator-activated receptor; SREBP: sterol regulatory element binding protein; TNF: tumor necrosis factor. This image has been created using BioRender.

Although the main trigger differs between the two pathologies (obesity, metabolic syndrome and insulin resistance being the main determinants of the development of NAFLD, and chronic heavy alcohol consumption in ArLD), an overlapping of common pathophysiological mechanisms has been demonstrated that explains the similar course of both diseases [143] (Figure 3).

Hepatic fat accumulation is a prerequisite for further inflammation. This is a consequence of an imbalance between the increased free fatty acid (FFA) influx (dietary sources, adipocytes and lipolysis mediated by alcohol), increased de novo lipogenesis in the liver, and decreased FFA oxidation and triglyceride exportation [144]. This imbalance is mainly mediated by the presence of systemic and liver inflammation, gut dysbiosis and genetic susceptibility among individuals.

Fatty liver activates a local immune response, which is the result of the interaction between activated macrophages and T-cells. This response is amplified through the secretion of several cytokines, such TNFα, IL-1β and IL-1. When this occurs, local immune response increases to a systemic immune activation in a pro-inflammatory state, which leads to liver damage. This mechanism is perpetuated in continuous feedback, increasing systemic inflammation. As a result, some transcription factors, such SREBP-1 and PPAR-α, are activated in hepatocytes, leading to de novo lipogenesis [119,120].

Another point of overlap in the pathophysiology is established by the gut microbiome. Recently, increased microbiome-mediated endogenous alcohol production has been demonstrated in patients with NASH [53,54,55,56,66]. This finding has been related to increased intestinal permeability and dysbiosis. In addition, increased intestinal permeability as a consequence of the direct toxic effects of alcohol has been demonstrated in ArLD. Furthermore, ethanol metabolites induce gut dysbiosis, leading PAMPs to reach the liver via systemic circulation and perpetuating the pro-inflammatory state within the liver [121]. Moreover, NAFLD- and ArLD-mediated dysbiosis may impact the enterohepatic circulation of bile acids, increasing serum and stool levels of secondary bile acids, which may be more potent activators of inflammation [145,146,147,148].

Finally, the last key element in pathogenesis common to both diseases is genetic factors. It has been demonstrated that people at risk of NAFLD are also at risk of ArLD based on individual heritable susceptibility. PNPLA3 has been associated (with the strongest correlation among other identified genes) to increased risk of steatosis, fibrosis and HCC in both NAFLD and ArLD [70,72,74,100,101]. TM6SF2 and MBOAT7 have also been related to the development of steatosis and fibrosis in both disorders. The tendency toward hepatic fat accumulation induced by these genetic variants is mediated by the alteration of several molecules implicated in the clearance and circulation of fatty acids. In a predisposed individual with hepatic fat accumulation, the apparition of any disorder that increases the levels of hepatic steatosis (such as NAFLD and ArLD patients) may induce a more severe fat accumulation, which leads to inflammation and liver damage [75,76,77,78,79,100]. Recently, the role of rs72613567 within hydroxysteroid 17-beta dehydrogenase 13 (HD17B13) in the development of liver disease, cirrhosis and HCC has been demonstrated to provide substantial protection from these disorders and a tendency towards decreased inflammation, reduced fibrosis and milder disease severity in patients with NAFLD.

Through these pathways, both NAFLD and ArLD will promote an imbalance between adaptative cell survival response and cell death, inducing the apoptosis of hepatocytes. In this setting, intracellular activation of interferon regulatory factor 3 (IRF3) [149] and stimulation of interferon gene protein (STING) triggers TANK-binding kinase (TBK) and activates mitochondrial apoptotic mechanisms in hepatocytes. This phenomenon will lead to a wound-healing response, and a marked fibrotic response will be developed, progressing to cirrhosis in the most severe cases [145,150,151].

### 4.2. Role of Alcohol Consumption in NAFLD

The definition of NAFLD requires a lack of significant alcohol consumption, which considers a maximum alcohol intake that is not well defined. Definition of this “significant” amount of alcohol varies in the literature from ≤1 drink (14 g of alcohol) to <30 g per day. However, other factors such as timing and duration of alcohol consumption and gender have not been considered when defining NAFLD. Taking this into account, there is no clear definition of “how much is too much” and what amount of alcohol consumption does not have deleterious effects on the liver in NAFLD patients [152,153,154,155,156].

Alcohol consumption frequently coexists with an excess in dietary caloric intake, suggesting its possible synergistic effect in liver damage. Moreover, patients with NAFLD are at increased risk of cardiovascular events. Emerging studies have provoked concerns about the possible benefits of light to moderate alcohol consumption in order to diminish this increased cardiovascular risk in patients with NAFLD. For these reasons, it is important to measure the impact of the effect of chronic alcohol consumption on patients with obesity and, therefore, in NAFLD.

Until now, in this context there is only clear evidence of the negative impact of chronic heavy alcohol consumption (>40 g per day, for years) and even moderate consumption (20–40 g of alcohol per day) in patients who are obese. In fact, alcohol consumption has been demonstrated to increase hepatic steatosis [90,157], inflammation [3,157,158], fibrosis [159], cirrhosis [157] and HCC [160,161,162] in overweight and/or obese patients. This evidence is summarized in Table 1.

In contrast, the negative effect of chronic moderate alcohol consumption in terms of progression of the disease and prognosis in patients with NAFLD has not been clearly demonstrated. This association has likely not been proven due to the heterogeneity of these studies and the inability to distinguish between patients with pure fatty liver and NASH [165,166], although some recent studies show a clear negative impact on the progression of liver disease [167]. Evidence of the impact of chronic alcohol consumption in NAFLD is summarized in Table 2.

Referring to the risk of HCC, the data of alcohol intake in patients who are overweight, obese or have NAFLD suggests there is an increased risk with any consumption of alcohol for the development of HCC in patients with NASH as reported in their liver histology [160,161,165,166].

### 4.3. Assessment of Alcohol Consumption in NAFLD Patients

The first step in reaching an ArLD diagnosis includes a search for signs of alcohol use disorder (AUD). This disorder is highly prevalent but poorly identified, likely because heavy alcohol consumption is difficult to detect [179]. AUD is defined by the presence of 2 or more of 11 diagnostic criteria in the past 12 months (Table 3).

A major clinical problem is determining the presence of early ArLD, including low or moderate ArSH, without any clinical symptoms. This scenario can lead to more advanced ArLD and subsequently a progression to cirrhosis, when signs of liver decompensation appear and the disorder becomes more evident but the prognosis is substantially worse.

Another issue is presented in the evaluation of patients with AUD. Often, these patients are treated by a psychiatrist; hepatic evaluation is not performed, and therefore early detection of ArLD is not possible.

In this context, current guidelines recommend that adults of 18 and older, including pregnant women, should be screened for unhealthy alcohol use in primary care settings, and those who engage in risky or hazardous drinking should receive behavioral counseling treatment to minimize unhealthy alcohol use [163]. Moreover, in patients with AUD and >40 g/day alcohol consumption, ArFL is present in 90–100% of individuals, and it is modulated by the presence of obesity [157,159,164] and the lack of abstinence. The former must be screened in all patients with ArLD, and when it is detected, patients must be provided with recommendations for eating habits and physical exercise. The latter must be suspected and screened in the clinical interview, but other clinical tools like biomarkers could be useful in this scenario [93].

Alcohol biomarkers are non-invasive tools for the assessment of recent or chronic alcohol consumption. Clinicians can determine several byproducts of alcohol metabolism in a few different samples (plasm, urine, hair, breath, i.e., direct biomarkers). The damage exerted by alcohol and its metabolites in several organs can also be measured using indirect biomarkers. These instruments provide useful information in many clinical scenarios (for example, diagnostic work-up of several liver diseases, follow-up in post-transplant liver recipients with previous alcohol consumption, evaluation for liver transplantation in patients with previous harmful alcohol use) not only for recent alcohol consumption (even with low amounts of alcohol intake) but also for chronic alcohol consumption. Table 4 summarizes the characteristics of the main biomarkers used in clinical settings for assessing alcohol consumption.

## 5. Conclusions

ArLD and NAFLD share common morphology and pathogenesis, including fatty liver as a prerequisite for the development of liver damage. Histological findings of NASH and ArSH are comparable and appear similar, suggesting the presence of common mechanisms in their pathophysiology. Multiple pathogenic pathways are present in both disorders and lead to liver damage, but the main trigger differs between ArLD and NAFLD. The former involves the direct toxic effects of ethanol and its metabolites as well as the dysregulation of fat metabolism directly derived from alcohol consumption. The latter involves enhanced fat metabolism pathways derived from the effect of excess calorie intake, insulin resistance and metabolic syndrome.

Those common mechanisms exert a synergistic effect in the development of liver damage and its progression to fibrosis, cirrhosis and HCC. Although some studies reveal a potential beneficial effect of light to moderate alcohol consumption in patients with NAFLD in terms of hepatic steatosis and insulin resistance improvement, most of those studies have potential bias, and their results should be considered with caution.

In the assessment of NAFLD patients, an extensive work-up must be done in order to detect the potential contribution of alcohol consumption in their prognosis. Moreover, referring to alcohol consumption in patients with NAFLD, clinicians must recommend avoiding any alcohol consumption, since potential cardiovascular and hepatic benefits at low doses of alcohol intake may be counteracted by the increased risk of neoplasia or AUD.

## Figures and Tables

**Table 1:** Evidence of the synergistic effect of alcohol consumption and obesity in liver-related outcomes. Abbreviations: ArLD: alcohol-related liver disease; AST: aspartate aminotransferase; BMI: body mass index; NASH: non-alcoholic steatohepatitis; OR: odds ratio.

Autor, Year	Outcomes	Design	Definition for Heavy Alcohol Consumption	Criteria for Overweight	Definition of NAFLD	Results
Naveau et al., 1997 [157]	Assess the impact of overweight in ALD	Retrospective cohort	>50 g/day of alcohol	BMI > 27 kg/m^2^ in men or >25 kg/m^2^ in women	Not defined	Higher risk in overweight patients of: Cirrhosis (60% vs. 35%; *p* = 0.001) Alcoholic hepatitis (8.2% vs. 2.6%; *p* = 0.05) Pure steatosis (8% vs. 2.5%; *p* = 0.05)
Bellentani et al., 2001 [90]	Characterization of liver diseases in general population	Prospective cohort	>30 g/day of alcohol	BMI > 25 kg/m^2^ in men or >24 kg/m^2^ in women	Not defined. Suspected by fatty liver in ultrasonography	Higher risk of hepatic steatosis in obese drinkers (>90%).
Raynard et al., 2002 [163]	Influence of alcohol in NASH patients	Prospective cohort	>50 g/day	Not defined	Liver biopsy	Association between duration of alcohol abuse and risk of significant fibrosis (*p* < 0.005)
Ruhl et al., 2005 [158]	Relationship between alcohol and overweight in patients with abnormal serum trasnaminases	Prospective cohort	>2 Drinks per day in overweight or >1 drink per day in obeses	BMI > 25 kg/m^2^	Not defined	Increased aminotransferase levels in obese and overweight (12% vs. 7.3% vs. 4.4%; *p* = 0.001)
Loomba et al., 2009 [164]	Association between alcohol, BMI, and liver enzymes	Cross-sectional	>30 g/day	BMI > 25 kg/m^2^	Not defined	Higher ALT and GGT levels. Increased risk of liver injury. Men OR 8.9 (95% CI, 2.4–33.1) Women OR 21-fold (95% CI, 2.6–170.1),
Eksted et al., 2009 [159]	Influence of alcohol intake in patients with NAFLD in terms of fibrosis stage progression	Retrospective cohort	Heavy episodic drinking (HED) (>60 g/day in male or >48 g/day in women at least 1 day in the past month)	BMI > 25 kg/m^2^	Liver biopsy	More fibrosis progression in HED (47% vs. 11%; *p* = 0.003)
Aberg et al, 2017 [164]	Metabolic factors implicated in the development of complicated liver disease	Retrospective cohort	>210 g/week men or >140 g/week women	BMI > 25 kg/m^2^	Metabolic syndrome	Higher incidence of complicated liver disease in drinkers OR 1.002 (1.001–1.002)

**Table 2:** Evidence of the effect of light to moderate alcohol consumption in the progression of liver disease in patients with NAFLD.

Author, Year	Primary Endpoint	Design	Sample Size	Criteria for NAFLD	Definition of Moderate Alcohol Use	Results	Bias
Alatalo et al., 2008 [168]	Link between alcohol consumption, BMI and liver enzymes	Retrospective cohort	457 Overweight 67 moderate alcohol use	-	<40 g/day	Higher serum ALT and GGT levels (*p* < 0.05)	NAFLD not defined
Cotrim et al., 2009 [169]	NASH on liver biopsy	Cross-sectional	132 NAFLD 75 moderate alcohol use	Liver biopsy	<40 g/day and <280 g/week	NASH more frequent among moderate alcohol users OR 2.69 (0.14–161.3) *p* = 0.41	Bariatric surgery population
Ekstedt et al., 2009 [159]	Fibrosis progression Development of cirrhosis	Prospective cohort	71 NAFLD 65 moderate alcohol use	Liver biopsy	<140 g/week	Higher risk of fibrosis progression for drinkers OR 7.11 (1.99–25.5) *p* = 0.003	Lifetime use not measured
Dunn et al., 2009 [170]	Prevalence of suspected NAFLD	Retrospective cohort	1031 NAFLD 523 moderate alcohol use	ALT > 43 or ALT > 30 men or ALT > 19 women	<10 g/day	Reduced prevalence of suspected NAFLD with alcohol OR 0.51 (0.33–0.79) *p* = 0.001	NAFLD not proven by liver biopsy
Gunji et al., 2009 [171]	Presence of fatty liver	Cross-sectional	5599 Fatty liver 2879 light to moderate alcohol use	Fatty liver on imaging test	Light alcohol use 40–140 g/week Moderate alcohol use 140–280 g/week	Light [OR 0.82 (0.63–0.94)] *p* = 0.044 Moderate [0.75 (0.61–0.92)] *p* = 0.008	NAFLD not proven by liver biopsy
Yamada et al., 2010 [172]	Presence of fatty liver	Cross-sectional	3127 fatty liver 2606 moderate alcohol use	Fatty liver on ultrasonography	<23 g/day	Daily moderate appeared protective 18.7% vs. 28.5% *p* = 0.05	NAFLD not proven by liver biopsy
Ascha et al., 2010 [160]	HCC on imaging	Prospective cohort	195 NAFLD 58 moderate alcohol use	Liver biopsy or cryptogenetic cirrhosis + metabolic syndrome	<2 drinks daily or 3–6 drinks daily on weekends	Higher risk of HCC for any alcohol use HR 3.8 (1.6–8.9) *p* < 0.002	Cirrhotic population
Moriya et al., 2011 [173]	Presence of fatty liver	Cross-sectional	2141 Fatty liver 677 moderate alcohol use	Fatty liver on imaging test	<20 g/day or <140 g/week	Low prevalence of fatty liver in moderate alcohol use OR 0.47 (0.23–0.96) *p* < 0.001	NAFLD not proven by liver biopsy
Dixon et al., 2011 [174]	NASH on liver biopsy	Cross-sectional	108 patients NAFLD 57 moderate alcohol use	Liver biopsy	<200 g/week	NASH less frequent among alcohol users OR 0.35 (0.12–1.0) *p* = 0.04 Not significant in multivariant	Morbidly obese
Hiramine et al., 2011 [175]	Prevalence of fatty liver	Cross-sectional	3816 fatty liver 1389 moderate alcohol use	Fatty liver on ultrasonography	<20 g/day & >21 days per month	Decreased prevalence of fatty liver in drinkers OR 0.55 [0.45, 0.67] *p* < 0.001	NAFLD not proven by liver biopsy
Hamaguchi et al., 2012 [176]	Presence of fatty liver and metabolic syndrome	Cross-sectional	4335 Fatty liver 937 moderate alcohol use	Fatty liver on ultrasonography	<280 g/week	Decreased prevalence of fatty liver in moderate alcohol use Men OR 0.72 (0.63–0.83) *p* < 0.001 Women 0.43 (0.21–0.88) *p* < 0.021	
Wong et al., 2012 [177]	Presence of NAFLD and fibrosis	Prospective cohort	264 fatty liver 148 moderate alcohol use	Liver fat and fibrosis assessed by proton-magnetic resonance and transient elastography	<10 g/day	Modest alcohol consumption not associated with fatty liver OR 1.37 (0.89–2.11); *p* = 0.15 Nor increased liver stiffness 2.3% vs. 1.7% *p* = 0.54	
Dunn et al., 2012 [170]	NASH progression (fibrosis stage)	Cross-sectional	582 NAFLD 331 moderate alcohol use	Liver biopsy	<20 g/day	Higher fibrosis stage among moderate alcohol users	Lifetime use not measured
Kwon et al., 2014 [178]	Advanced fibrosis (stage 3–4)	Cross-sectional	77 NAFLD 52 moderate alcohol use	Liver biopsy	<40 g/week	Less risk of advanced fibrosis among drinkers OR 0.26 (0.07–0.97) *p* = 0.046	Alcohol pattern not determined

**Table 3:** Definition of alcohol use disorder (AUD). The presence of at least two symptoms indicates AUD. Mild: 2–3 symptoms. Moderate: 4–5 symptoms. Severe: 6 or more symptoms.

	Your Experience Last Year
1	Alcohol is often taken in largen amounts or over a longer period than intended
2	There is a persistent desire or unsuccessful efforts to cut down or control alcohol use
3	A great deal of time is spent in activities necessary to obtain alcohol, use alcohol, or recover from its effects
4	Craving, or a strong desire or urge to use alcohol
5	Recurrent alcohol use resulting in a failure to fulfill major role obligation at work, school, or home.
6	Continued alcohol use despite having persistent or recurrent social or interpesonal problems caused or exacerbated by the effects of alcohol
7	Important social, occupational, or recreational activities are given up or reduced because of alcohol use.
8	Recurrent alcohol use in situation in which it is physically hazardaous.
9	Alcohol use is continued despite knowledge of having a persistent or recurrent physical or psychological problem that is likely to have been caused or exacerbated by alcohol
10	Tolerance, defined as either of the following A. Need for markedly increased amounts of alcohol to achieve intoxication or desired effect; or B. Markedly diminished effect with continued use of the same amount of alcohol.
11	Withdrawal, as manifested by either of the following: A. The characteristics alcohol withdrawal syndrome; or B. Alcohol (or a closely related substance, such as benzodiazepine) is taken to relieve or avoid withdrawal symptoms.

**Table 4:** Alcohol biomarkers.

	Concept	Pattern	Sample	Window	Pros	Cons
**Indirect**						
>1.5–2 AST/ALT ratio	Mitochondrial damage by alcohol	Chronic heavy drinking (>40 g/day).	Serum or plasm	2–3 weeks after consumption	Cheap Accesible	Lack of sensitivity and specificity
GGT	Glutation metabolism	Chronic heavy drinking (>40 g/day).	Serum or plasm	2–3 weeks after consumption	Cheap Accesible Higher values can predict steatohepatitis	Lack of sensitivity and specificity
MCV	Toxic effect of acetaldehyde in morphology of red blood cells	Chronic heavy drinking (>40 g/day).	Serum or plasm	2–8 weeks after consumption Normalization after 2–4 months of abstinence	Cheap Accessible	Altered in hematological diseases
CDT	Deficient binding of carbohydrates to trasnferrin in presence of alcohol	Chronic heavy drinking (>40 g/day).	Serum or plasm	2–4 weeks after consumption Normalization within 2–4 weeks of abstinence	Best sensitivity and specificity of indirect biomarkers	Not widely available
**Direct**						
5-HTOL	Ehtanol adducts with hydroxytryptophol	Recent consumption (>20 g/day)	Urine	< 24 h	Quick Highly specific (99%)	Low sensitivity Not widely available Not for chronic alcohol use
PEth	Ethanol adducts with phospholipids	Chronic consumption (>30–40 g/day)	Dry drop	2–4 weeks after consumption Normalization within 2 weeks of abstinence	Cheap Quick Accessible	Not widely available
EtG	Ethanol adducts with glucuronide	Acute or chronic consumption (>10 g/day)	Blood (<36 h), urine (<96 h) and hair (months)		High specificity and sensitivity near 100%	False positives in CKD and THC consumption
FAEEs	Ethanol adducts with fatty acids	Acute or chronic consumption	Plasm (<24–96 h) and hair (months)		Highly specific	Low sensitivity Not widely available

## Data Availability

No new data were created or analyzed in this study. Data sharing is not applicable to this article.
